# Healthcare cost expenditures associated to frailty and sarcopenia

**DOI:** 10.1186/s12877-022-03439-z

**Published:** 2022-09-13

**Authors:** Alejandro Álvarez-Bustos, Beatriz Rodríguez-Sánchez, Jose A. Carnicero-Carreño, Walter Sepúlveda-Loyola, Francisco J. Garcia-Garcia, Leocadio Rodríguez-Mañas

**Affiliations:** 1 Biomedical Research Center Network for Frailty and Healthy Ageing (CIBERFES), Institute of Health Carlos III, Madrid, Spain; 2 Department of Applied Economics, Public Economics and Political Economy, Faculty of Law, University Complutense of Madrid, Madrid, Spain; 3 Biomedical Research Foundation, Getafe University Hospital, Getafe, Spain; 4 Faculty of Health and Social Sciences, Universidad de Las Americas, Santiago, Chile; 5 Masters and PhD Programme in Rehabilitation Sciences, Londrina State University (UEL) and University North of Paraná (UNOPAR), Londrina, Brazil; 6 Geriatrics Department, Virgen del Valle Hospital, Toledo, Spain; 7 Geriatrics Department, Hospital Universitario de Getafe, Getafe University Hospital, Ctra de Toledo km 12,500, 28905 Getafe, Spain

**Keywords:** Frailty, Sarcopenia, Hospital admission, Healthcare costs, Ageing

## Abstract

**Objectives:**

Frailty and sarcopenia have been related with adverse events, including hospitalization. However, its combined effect with hospitalization-related outcomes, including costs, has not been previously investigated. Our purpose was to explore how frailty, sarcopenia and its interaction could impact on healthcare expenditures.

**Methods:**

1358 community-dwelling older adults from the Toledo Study of Healthy Ageing (TSHA) were included. Sarcopenia was measured using the Foundation for the National Institutes of Health criteria fitted to our cohort. Frailty was defined according to Frailty Trait Scale 5 (FTS5) and the Frailty Index fitted to the cut-off points of TSHA population. Hospitalization costs were taken from hospital records and costs were attributed according to Diagnostic-Related Groups, using as the cost base year 2015. Two-part regression models were used to analyze the relationship between frailty and sarcopenia and hospital admission, number of hospitalizations, length of stay and hospitalization costs.

**Results:**

Sarcopenia was associated only with the probability of being admitted to hospital. Frailty was also associated with higher hospital use, regardless of the frailty tool used, but in addition increased hospital admission costs at follow-up by 23.72% per year and by 19.73% in the full model compared with non-frail individuals. The presence of sarcopenia did not increase the costs of frailty but, by opposite, frailty significantly increased the costs in people with sarcopenia, reaching by 46–56%/patient/year at follow-up. Older adults with frailty and sarcopenia had a higher risk of hospitalization, disregarding the tool used to assess frailty, and higher hospitalization costs (FTS5) in the full model, at the cross-sectional and at the follow-up level.

**Conclusions:**

Frailty is associated with increased hospitalization costs and accounts for the potential effects of sarcopenia.

**Supplementary Information:**

The online version contains supplementary material available at 10.1186/s12877-022-03439-z.

## Introduction

Healthcare systems are suffering an increasing burden in terms of a heavier demand due to chronic health diseases accompanying the ageing of the population. Since 1980, people aged 65 years and older has almost tripled, and prospective studies expected these population to double by 2050 [1]. Although healthcare expenditures (HCE) increase as people age, age itself is considered a distraction from the true drivers of spending [2, 3], like physical debilitating conditions [4]. This is also true for those who are not heavily disabled: older adults with moderate functional limitations are expected to live for longer periods of time and with greater use of medical care than most older adults with disabilities, increasing in an exponential way as their frailty status worsen [5].

Frailty and sarcopenia, two highly prevalent conditions in older people, are characterized by physical function impairment with a high potential risk of disability [6, 7]. Frailty is defined as an age-associated, biological syndrome characterized by decreased biological reserves of the individual, increasing the risk of adverse events when facing minor stressors [7]. On the other hand, sarcopenia is defined as a loss of lean mass, strength and/or function usually associated with ageing [8]. These entities are closely related to chronic disease, a high prevalence of polypharmacy [9, 10] and adverse events, such as falls and poor quality of life [11–13] In addition, it has been proposed that older people with frailty or sarcopenia are more likely to be hospitalized, augmenting health service costs [4, 14–16]. Furthermore, frailty and sarcopenia could be reverted [17–20], and the cost associated to them avoidable, thus preventing imminent future society demands.

Frailty and sarcopenia have been related between them [21], could coexist, but are two different conditions [22]. In addition, the lack of a universal gold standard for frailty assessment has allowed a wide spectrum of frailty assessment tools that focus on different characteristics of subjects with a poor agreement between them [23–25]. The Frailty Index is one of the most used frailty tools inside the “cumulative deficit” approach to frailty. It is constructed through a long checklist of symptoms, signs, diseases, disabilities, laboratory, radiographic or electrocardiographic abnormalities and social characteristics [26, 27] and even health utilization [28]. The Frailty Trait Scale 5 (FTS5) is an instrument stemming from the frailty phenotype conceptual framework [13] with a predictive ability of adverse events in community-dwelling older adults that could improve the FI one and aiming to overcome some of its drawbacks [29]. It includes objective functional measures, and it has proven to be highly dynamic and sensitive to changes with different risks of adverse events as mortality, hospitalization and disability associated to its score [30]. Although there are some studies that have already supported the increasing economic burden of frailty and sarcopenia in terms of healthcare costs in older populations [4, 31–34], to the best of our knowledge, there is no study that has analyzed the economic impact of these two prevalent conditions separately and their interaction on costs related to hospitalizations in older adults. This gap in the literature has motivated the purpose of this study, motivating the purpose of our research.

## Methods

Participant data were taken from the Toledo Study of Healthy Ageing (TSHA). TSHA is a longitudinal cohort that was designed to analyze different models of frailty and ageing and to measure the impact that frailty, comorbidity and disability may have on health system by assessing rural and urban community dwelling older adults aged 65 years or older [35]. TSHA was performed in concordance with the Helsinki Declaration of 1975 and was approved by the Clinical Research Ethics Committee of the Toledo Hospital in Spain. Participants signed an informed consent form. Visits were performed in the second wave of the study (basal visit) between 2011 and 2013 and the outcomes were collected during the third wave (until December 2015).

### Measures of frailty

Frailty was measured using Frailty Index (FI) and Frailty Trait Scale 5 (FTS5).

Frailty Index: characterizes frailty as an accumulation of deficits. It is based on the ratio between deficits present in the individual and the total measured deficits [36]. Subjects were considered frail if the score was ≥0.275. The TSHA FI was built following Searle et al., using 40 items with scores of 0 to 1 [37]. TSHA FI scoring tool is displayed in the Supplementary Information Table S1.

Frailty Trait Scale 5 [29]: a Short Form of the 12 items one [38] is a continuous scale that monitor frailty status in a range between 0 to 50. Each domain score range between 0 to 10. Subjects were considered as frail if their score were > 25 and no frail ≤25 [29]:

- Physical Activity: defined using the PASE scale [39].
- Gait speed: was defined using the 3-m walking test at their usual pace, according to the standard protocol. Best time of two performances was chosen.
- Hand grip strength: was measured using JAMAR Hydraulic Hand Dynamometer (Sammons Preston Rolyan, Bolingbrook, IL). Best peak strength of three performances was selected and gathered using international standard procedures [40]. Between performances, at least 1 min of resting was permitted.
- Body Mass Index: was measured according to the standard recommendation (weight/height^2^).
- Balance test: was stablished according to the standing balance feet together, semi-tandem, and tandem position (progressive Romberg balance test) [41].

FTS5 scoring tool is displayed in the Supplementary Information Table S2.

### Sarcopenia

Sarcopenia was measured at baseline and was calculated according to the Foundation for the National Institutes of Health (FNIH), but fitted to the cut-points of our population (standardized FNIH [sFNIH]) [22]. According to this definition, sarcopenia is present when the low grip strength, low muscle mass and low gait speed criteria are met.

Muscle mass was determined using Dual-Energy X-ray Absorptiometry (DEXA) (Hologic, Serie Discovery QDR, Bedford, MA, USA). DXA scans were analyzed using the software Physician’s Viewer (apex System Sofware, version 3.1.2: Bedford, USA). BMI- adjusted by Appendicular Lean Mass (ALM/BMI), derived as the sum of the muscle mass of the arms and legs, it was used as marker of low lean mass. According to sFNIH diagnosis algorithm, low muscle mass was considered in men and women when ALM/BMI is below 0.65 and 0.54 respectively.

Gait speed and handgrip strength measurement methodologies were explained above. Gait speed cut-off point was < 0.8 m/s and handgrip strength cut-off points were < 25.51 kg for men and < 19.19 kg for women.

### Hospitalization

Hospitalization after recruitment was defined as first admission and was registered reviewing the records of Toledo Hospital Complex. Participants’ hospitalizations were followed-up to December 2015 with a median of 167 weeks (36.43 weeks SD).

Hospitalization is composed of four measures: having been admitted to hospital which takes value 1 if the respondent has been hospitalized in the previous 12 months and 0 otherwise, number of hospitalizations per year, average length of stay in days per year and hospitalization costs in euros per year. In order to estimate hospitalization costs, we used data from each survey respondent at baseline and during the follow-up period from hospital clinical records and we subsequently estimated the costs per person and per year in €. The unit costs per each Diagnostic Related Group (DRG), which were obtained from national data sources, were multiplied by the number of hospital admissions for each DRG. All costs were updated, if applicable, and expressed in 2015 euros.

### Covariates

Age and gender were self-reported. Comorbidities were ascertained by the Charlson Index Score [42]. Polypharmacy was defined as the intake of ≥5 drugs/day [43]. All covariates were measured at the baseline visit (between 2011 and 2013).

### Study variable and statistical analysis

Given the substantial proportion of zeros within the number and costs of hospital admission, as well as the length of stay, which led to a skewed distribution of the data, two-part regression models will be run for such outcomes, which assume a first part for modelling the probability of being admitted to hospital and a second part on the positive continuous outcome only on those who have been admitted to hospital.

Two-part models combine a model for the binary response variable, which would take value 1 if the individual has been admitted to hospital at least once and 0 if no hospitalizations, and a model for the outcome variable that is conditioned on the binary response [44], conditional on having been admitted to hospital.

The first stage of the two-part model was performed as a traditional logit regression model, in which the estimated coefficients capture the effects on the log-odds-ratio [45].

The second stage involves a Generalized Linear Model (GLM) with a gamma distribution in case of hospitalization costs with a log link and a zero-truncated Poisson distribution for the number of hospitalizations and length of stay, in days.^1^ GLM models have frequently been used for healthcare costs analysis recently, given the skewed distribution of costs [47]. GLMs are empirical transformations of the classical ordinary least square (OLS) regression model, which specify the conditional mean function directly. Specifically, GLMs do not require transformation scales, but a response distribution of one of the exponential family of distributions (normal, Poisson, gamma, binomial, inverse Gaussian), which relates the mean of the response to a scale on which the model effects combine additively [48]. As previously stated, according to the Modified Park Test, the chosen family was the Gamma distribution for modelling total hospitalization costs and the Poisson distribution for the other two continuous outcomes in our analysis [49].

Several regression models have been run. The first regression model only includes one of the main variables of interest (sarcopenia or frailty). In a second regression model, age and its square, and gender have been included. Model three adds to Model two the other variable of interest (sarcopenia or frailty, as applicable). Models four adds the comorbidity severity of individuals according to the Charlson Index, which is medium-low if the Charlson Index score is 1 or 2; and high if the Charlson Index score is three or higher. Moreover, polypharmacy is also included if the daily number of drugs the subject is taking is 5 or more. We also perform the likelihood ratio test in order to detect overfitting after the inclusion of new variables in the nested models.

Two assessments will be made regarding the association between frailty and sarcopenia and hospitalization outcomes: the first analysis will entail the associations within the same wave (outcome and main variables of interest measured in wave 2), whereas the second analysis will consider outcomes at follow-up, wave 3, and the independent variables evaluated in wave 2. The sample size will be reduced at follow-up assessment from 1358 individuals to 1349 older adults due to mortality, during the hospital admission (*n* = 9).

## Results

Participants´ characteristics classified depending on frailty status according to different frailty tools and sarcopenia are shown in Table 1. Prevalence of frailty are 12.42% (FI) and 10.38% (FTS5). In our sample, 22.31% were diagnosed with sarcopenia.

**Table 1:** Characteristics of the sample, at baseline

Variable	Frailty Index			FTS5			Sarcopenia		
	Frail	No frail	*p*-value	Frail	No frail	*p*-value	With sarcopenia	Without sarcopenia	*p*-value
N (%)	83 (6.11)	1275 (93.89)		141 (10.38)	1217 (89.62)		303 (22.31)	1055 (77.69)	
Age (SD)	81.72 (5.65)	74.73 (5.89)	< 0.001***	79.48 (5.76)	74.02 (5.47)	< 0.001***	77.5**3** (5.5**2**)	73.7**4** (5.5**2**)	< 0.001***
Gender, female, **n** (%)	**936** (68.90)	**718** (52.85)	< 0.001***	**1021** (75.18)	**693** (51.03)	< 0.001***	**1116** (82.18)	**615** (45.31)	< 0.001***
Sarcopenia yes, **n** (%)	**867** (63.86)	**266** (19.61)	< 0.001***	**1088** (80.14)	**212** (15.61)	< 0.001***	–	–	–
Charlson Index (SD)	2.25 (2.30)	1.08 (1.46)	< 0.001***	1.76 (1.93)	1.11 (1.54)	< 0.001***	1.5**7** (1.9**0**)	1.0**6** (1.4**8**)	< 0.001***
Drugs, mean (SD)	7.25 (3.40)	4.74 (2.86)	< 0.001***	6.95 (2.86)	4.56 (2.77)	< 0.001***	6.1**8** (2.9**7**)	4.4**2** (2.7**3**)	< 0.001***
Polypharmacy, n (%)	168 (80.38%)	731 (49.59%)	< 0.001***	112 (79.43%)	581 (47.74%)	< 0.001***	303 (69.97%)	1055 (45.59%)	< 0.001***
Hospitalization yes, **n** (%)	**370** (27.27)	**117** (8.62)	< 0.001***	**221** (16.31)	**108** (7.97)	0.001***	**166** (12.21)	**107** (7.88)	0.019*

Mean (SD): continuous variables. N, %: categorical variable

*FTS5* Frailty Trait Scale 5

*** *p* < 0.001, ** *p* < 0.01, * *p* < 0.05

Regardless of the tool used to assess frailty, in the bivariant analyses frail individuals seem to be older, more likely to have sarcopenia, and to be in worse health status (higher Charlson index value). Moreover, they seem to be more prone towards polypharmacy. Older adults with sarcopenia presented similar characteristics to those with frailty.

Table 2 reports the differences in hospitalization-related outcomes, showing a higher number of hospital admissions, longer stays, and higher hospitalization costs among older adults with frailty and with sarcopenia, compared to their counterparts.

**Table 2:** Summary statistics on hospitalization-related outcomes, at baseline

Variable	Frailty according to the Frailty Index		Frailty according to the FTS5		Sarcopenia	
	Frail	No frail	Frail	No frail	With sarcopenia	Without sarcopenia
Number of hospitalizations						
P25	0	0	0	0	0	0
P50	0	0	0	0	0	0
P75	1	0	1	0	0	0
P95	3	1	2	1	2	1
Average length per hospital admission						
P25	0	0	0	0	0	0
P50	0	0	0	0	0	0
P75	7.75	0	0.25	0	0	0
P95	**29.50**	**7.50**	18.25	**6.50**	**10.50**	6
Accumulated costs of hospital admission, 2015€						
P25	0	0	0	0	0	0
P50	0	0	0	0	0	0
P75	1279.36	0	517.51	0	0	0
P95	4782.86	2058.84	2323.29	1939.79	2264.69	1884.10

*FTS5* Frailty Trait Scale 5

### Results from the regression analyses

Table 3 shows that sarcopenia, when it is the only independent variable, is associated with an increased risk of being admitted to hospital both at the cross-sectional level (OR 1.56) and at follow-up (OR 1.47). However, it is not significantly related to the other hospital-related outcomes. Such associations were observed in the second regression model, when age and gender were considered (OR 1.48 in the cross-sectional analysis and OR 1.76 at follow-up), but only remained at follow-up when frailty entered the analysis (Model 3). In the fourth regression model, sarcopenia was only significantly associated with higher odds of being admitted to hospital at follow-up (OR 1.46) when frailty is evaluated by the Frailty Index.

**Table 3:** Results on the association between sarcopenia and the hospitalization-related outcomes, both at the cross-sectional level and at-follow up

	Cross-sectional analysis		At follow-up	
	Sarcopenia if frailty is measured via the Frailty Index	Sarcopenia if frailty is measured via the FTS5	Sarcopenia if frailty is measured via the Frailty Index	Sarcopenia if frailty is measured via the FTS5
Model 1				
OR on the probability of being admitted to hospital	1.557** (1.141–2.106)		1.469** (1.133–1.982)	
Coeff on number of hospital admissions	0.221 (− 0.358–0.799)		0.009 (− 0.366–0.384)	
Coeff on length of hospitalization	0.162 (− 0.221–0.545)		− 0.037 (− 0.343–0.269)	
Coeff on hospitalization costs	− 0.032 (− 0.320–0.255)		− 0.063 (− 0.295–0.168)	
Model 2				
OR on the probability of being admitted to hospital	1.481* (1.034–2.104)		1.760*** (1.283–2.456)	
Coeff on number of hospital admissions	0.513 (− 0.271–1.298)		−0.011 (− 0.484–0.463)	
Coeff on length of hospitalization	0.159 (− 0.307–0.626)		− 0.140 (− 0.531–0.250)	
Coeff on hospitalization costs	0.055 (− 0.248–0.359)		− 0.051 (− 0.303–0.202)	
Model 3				
OR on the probability of being admitted to hospital	1.318 (0.909–1.895)	1.456 (0.991–2.134)	1.629** (1.177–2.301)	1.477* (1.028–2.132)
Coeff on number of hospital admissions	0.484 (− 0.387–1.355)	0.387 (− 0.507–1.281)	− 0.142 (− 0.669–0.385)	−0.231 (− 0.642–0.180)
Coeff on length of hospitalization	− 0.033 (− 0.565–0.498)	−0.096 (− 0.599–0.406)	−0.298 (− 0.726–0.129)	−0.244 (− 0.705–0.216)
Coeff on hospitalization costs	0.025 (− 0.282–0.333)	0.122 (− 0.227–0.471)	−0.093 (− 0.359–0.172)	−0.129 (− 0.374–0.116)
Model 4				
OR on the probability of being admitted to hospital	1.197 (0.824–1.726)	1.318 (0.897–1.934)	1.463* (1.059–2.074)	1.348 (0.941–1.944)
Coeff on number of hospital admissions	0.357 (− 0.404–1.117)	0.264 (−0.533–1.061)	−0.180 (− 0.654–0.293)	−0.270 (− 0.637–0.097)
Coeff on length of hospitalization	−0.092 (− 0.558–0.374)	−0.151 (− 0.585–0.283)	−0.035 (− 0.747–0.045)	−0.318 (− 0.735–0.099)
Coeff on hospitalization costs	0.013 (− 0.283–0.309)	0.091 (− 0.241–0.423)	−0.126 (− 0.373–0.122)	−0.164 (− 0.403–0.075)

95% confidence intervals in parentheses. *** *p* < 0.001, ** *p* < 0.01, * *p* < 0.05

Model 1 includes sarcopenia as the only independent variable. Model 2 enters sociodemographic characteristics (age and its square and gender) in addition to sarcopenia. Model 3 adds to Model 2 the frailty status, measured by the Frailty Index or the Frailty Trait Score. Models 4 adds the comorbidity severity of individual according to the Charlson Index, which is medium-low if the Charlson Index score is 1 or 2; and high if the Charlson Index score is three or higher. Moreover, polypharmacy is also included if the daily number of drugs the subject is taking is 5 or more

As it is shown in the Table 4, in the raw model, frailty was cross-sectionally associated with higher risk of hospitalization, mean length of stay (the log-count of average length of stay increases by 0.74 and 0.53 days in case of being frail according to the FI and the FTS5, respectively, compared to non-frail older adults), disregarding the tools used, and with an increase in hospital costs by 22.85%^2^ when FTS-5 was used to assess the condition, but not when FI was used. In the fully adjusted model, only FI showed a significantly higher risk of hospitalization. When the association in the follow-up was estimated, frailty was associated with almost all the outcomes. Frailty was associated with an increase in costs, ranging from an increase by 13.98% (FTS-5, Model 2) to a maximum increase by 38.69% (FI, Model 1). When fully adjusted, some of the figures and associations changed. When we assessed the relationship between frailty and the outcomes in the long-term, we observed an association between frailty and the probability of being admitted to hospital (FTS5), the number of hospital admissions (FTS5 and FI) and length of hospitalization (FI, which increased the log count of average length of stay by 0.611 days). The increase in costs was only significant for the FTS5, which were higher by 19.73% among frail individuals than the non-frail. The differences in the significant associations between frailty and hospital admission and sarcopenia and hospital admission observed before were confirmed in Table 5, when the different combinations of sarcopenia and frailty status entered the analysis. Older adults with sarcopenia but without frailty showed a significantly higher probability of hospitalization at follow-up regardless of the tool used in Models 2 and 3, but not for the other outcomes either cross-sectional or longitudinal. On the other hand, older adults with frailty but without sarcopenia were significantly more likely to have most of the hospitalization-related outcomes in the raw model, especially when frailty was assessed by the Frailty Index, although only the length of hospital stay was significantly longer in the fully adjusted cross-sectionally model, and number and length of hospitalization for the FI at follow-up. However, older adults that have frailty and sarcopenia presented a significant association with the likelihood of being admitted to hospital, both at the cross-sectional and the follow-up levels, regardless of the frailty tool when the interaction was the only variable. In the fully adjusted model, both tools showed significant association with the probability of being admitted to hospital, in both cross-sectional and longitudinal analysis, but only FTS5 showed higher hospitalization costs, which increased by 11.18 and 4.74% among frail individuals with sarcopenia against the reference category, at the cross-sectional and at follow-up level, respectively.^3^ Additionally, we assessed the likelihood ratio test for each regression model in order to estimate if the sequence of models suffer from overfitting (Table 6).

**Table 4:** Results on the association between frailty and the hospitalization-related outcomes, both at the cross-sectional level and at-follow up

	Cross-sectional analysis		At follow-up	
	Frailty measured with the Frailty Index	Frailty measured with the FTS5	Frailty measured with the Frailty Index	Frailty measured with the FTS5
Model 1				
OR on the probability of being admitted to hospital	3.611*** (2.666–4.899)	1.586* (1.045–2.361)	2.687*** (1.990–3.609)	2.127*** (1.505–3.110)
Coeff on number of hospital admissions	0.366 (− 0.092–0.824)	0.397 (− 0.247–1.039)	0.535*** (0.306–0.764)	0.327* (0.104–0.757)
Coeff on length of hospitalization	0.739*** (0.417–1.061)	0.526* (0.094–0.959)	0.626*** (0.349–0.902)	0.183* (0.062–0.360)
Coeff on hospitalization costs	0.104 (− 0.121–0.330)	0.206* (0.075–0.487)	0.212* (0.040–0.385)	0.064 (− 0.239–0.367)
Model 2				
OR on the probability of being admitted to hospital	2.797*** (2.000–3.923)	1.283* (1.003–1.996)	2.337*** (1.677–3.221)	2.086*** (1.420–3.181)
Coeff on number of hospital admissions	0.558 (− 0.081–1.196)	0.515 (− 0.178–1.209)	0.628*** (0.342–0.914)	0.397* (0.084–0.877)
Coeff on length of hospitalization	0.845** (0.349–1.344)	0.498* (0.039–0.958)	0.724** (0.303–1.145)	0.155 (− 0.241–0.551)
Coeff on hospitalization costs	0.168 (− 0.062–0.398)	0.112* (0.039–0.185)	0.327** (0.142–0.512)	0.131* (0.013–0.249)
Model 3				
OR on the probability of being admitted to hospital	2.172** (1.296–3.666)	1.054 (0.643–1.697)	1.766* (1.065–2.876)	1.701* (1.102–2.716)
Coeff on number of hospital admissions	0.127 (− 0.717–0.972)	0.301 (− 0.470–1.073)	0.616** (0.189–1.043)	0.518* (0.069–0.966)
Coeff on length of hospitalization	0.719** (0.183–1.256)	0.555* (0.068–1.042)	0.761** (0.291–1.231)	0.274 (− 0.202–0.750)
Coeff on hospitalization costs	0.179 (− 0.214–0.572)	0.186 **(− 0.143–0.229)**	0.278* (0.003–0.553)	0.201* (0.115–0.287)
Model 4				
OR on the probability of being admitted to hospital	1.801* (1.070–3.046)	0.937 (0.569–1.523)	1.445 (0.870–2.360)	1.481* (1.014–2.403)
Coeff on number of hospital admissions	− 0.101 (− 0.819–0.617)	0.183 (− 0.516–0.882)	0.509* (0.075–0.944)	0.473* (0.066–0.881)
Coeff on length of hospitalization	0.450 (− 0.016–0.916)	0.357 (− 0.075–0.789)	0.611** (0.200–1.023)	0.220 (− 0.210–0.650)
Coeff on hospitalization costs	0.008 (− 0.342–0.359)	0.212 **(− 0.113–0.538)**	0.227 (− 0.060–0.514)	0.181* (0.012–0.350)

95% confidence intervals in parentheses. *** *p* < 0.001, ** *p* < 0.01, * *p* < 0.05

Model 1 includes frailty status, measured by the Frailty Index or the Frailty Trait Score, as the only independent variable. Model 2 enters sociodemographic characteristics (age and its square and gender) in addition to frailty. Model 3 adds to Model 2 sarcopenia. Models 4 adds the comorbidity severity of individual according to the Charlson Index, which is medium-low if the Charlson Index score is 1 or 2; and high if the Charlson Index score is three or higher. Moreover, polypharmacy is also included if the daily number of drugs the subject is taking is 5 or more

**Table 5:** Results on the association between the frailty and sarcopenia status and the hospitalization-related outcomes, both at the cross-sectional level and at-follow up

	Cross-sectional analysis		At follow-up	
	Frailty measured with the Frailty Index	Frailty measured with the FTS5	Frailty measured with the Frailty Index	Frailty measured with the FTS5
Model 1				
*Non-frail #* without sarcopenia	*Ref.*	*Ref.*	*Ref.*	*Ref.*
*Non-frail #* with sarcopenia				
OR on the probability of being admitted to hospital	1.295 (0.908–1.828)	1.388 (0.949–2.030)	1.357 (0.991–1.871)	1.293 (0.916–1.827)
Coeff on number of hospital admissions	0.258 (− 0.443–0.959)	0.101 (− 0.683–0.884)	− 0.095 (− 0.618–0.429)	− 0.335 (− 0.728–0.059)
Coeff on length of hospitalization	0.264 (− 0.185–0.713)	0.007 (− 0.452–0.466)	−0.125 (− 0.487–0.238)	−0.122 (− 0.527–0.282)
Coeff on hospitalization costs	−0.005 (− 0.327–0.317)	0.043 (− 0.341–0.427)	− 0.096 (− 0.377–0.185)	−0.177 (− 0.429–0.075)
*Frail #* without sarcopenia				
OR on the probability of being admitted to hospital	2.926** (1.367–6.262)	1.334 (0.533–3.339)	2.771** (1.333–5.760)	3.175** (1.492–6.754)
Coeff on number of hospital admissions	0.391 (− 0.694–1.477)	0.467 (− 0.311–1.245)	0.733** (0.241–1.226)	0.165 (− 0.561–0.891)
Coeff on length of hospitalization	1.302*** (0.745–1.859)	0.891** (0.261–1.521)	0.985** (0.371–1.599)	0.337 (− 0.510–1.184)
Coeff on hospitalization costs	0.380 (− 0.240–1.001)	0.362* (0.070–0.795)	0.259 (− 0.207–0.726)	−0.045 (− 0.579–0.490)
*Frail #* with sarcopenia				
OR on the probability of being admitted to hospital	3.316*** (1.854–5.931)	1.768* (1.113–2.740)	2.621*** (1.485–4.624)	2.025*** (1.376–3.116)
Coeff on number of hospital admissions	0.232 (− 0.568–1.031)	0.406 (−0.343–1.154)	0.443* (0.052–0.834)	0.307 (− 0.194–0.807)
Coeff on length of hospitalization	0.424 (− 0.146–0.994)	0.436 (−0.080–0.951)	0.458* (0.038–0.878)	0.101 (− 0.287–0.488)
Coeff on hospitalization costs	−0.005 (− 0.505–0.496)	0.169* (0.144–0.194)	0.093 (− 0.218–0.403)	0.057 (−0.296–0.411)
Model 2				
*Non-frail #* without sarcopenia	*Ref.*	*Ref.*	*Ref.*	*Ref.*
*Non-frail #* with sarcopenia				
OR on the probability of being admitted to hospital	1.324 (0.895–1.941)	1.427 (0.943–2.162)	1.681** (1.200–2.411)	1.640* (1.119–2.395)
Coeff on number of hospital admissions	0.567 (−0.340–1.474)	0.401 (− 0.579–1.380)	−0.100 (− 0.737–0.536)	−0.340 (− 0.795–0.115)
Coeff on length of hospitalization	0.314 (− 0.229–0.857)	0.034 (− 0.479–0.547)	−0.203 (− 0.661–0.255)	−0.211 (− 0.691–0.269)
Coeff on hospitalization costs	0.078 (− 0.248–0.405)	0.111 (− 0.271–0.493)	−0.085 (− 0.374–0.203)	−0.158 (− 0.425–0.109)
*Frail #* without sarcopenia				
OR on the probability of being admitted to hospital	2.216 (0.994–4.945)	0.940 (0.361–2.448)	2.109 (0.977–4.545)	2.785* (1.187–6.540)
Coeff on number of hospital admissions	0.416 (− 0.823–1.655)	0.363 (−0.778–1.504)	0.687* (0.135–1.240)	0.321 (− 0.410–1.053)
Coeff on length of hospitalization	1.256*** (0.716–1.796)	0.945** (0.380–1.510)	0.918** (0.250–1.585)	0.349 (−0.538–1.235)
Coeff on hospitalization costs	0.379(− 0.205–0.962)	0.258* (0.131–0.385)	0.312 (− 0.139–0.763)	0.100 (−0.410–0.610)
*Frail #* with sarcopenia				
OR on the probability of being admitted to hospital	2.837*** (1.501–5.370)	1.561 (0.933–2.553)	2.638** (1.428–4.847)	2.305*** (1.495–3.726)
Coeff on number of hospital admissions	0.518 (− 0.389–1.424)	0.682 (−0.196–1.559)	0.437* (0.014–0.860)	0.316 (− 0.275–0.906)
Coeff on length of hospitalization	0.428 (− 0.202–1.057)	0.400 (−0.189–0.990)	0.371 (− 0.102–0.843)	0.014 (− 0.428–0.456)
Coeff on hospitalization costs	0.114 (−0.390–0.618)	0.054* (0.001–0.107)	0.167 (− 0.154–0.488)	0.091 (−0.271–0.452)
Model 3				
*Non-frail #* without sarcopenia	*Ref.*	*Ref.*	*Ref.*	*Ref.*
*Non-frail #* with sarcopenia				
OR on the probability of being admitted to hospital	1.170 (0.786–1.729)	1.252 (0.820–1.911)	1.483* (1.057–2.144)	1.469* (1.002–2.149)
Coeff on number of hospital admissions	0.401 (−0.437–1.240)	0.255 (− 0.664–1.174)	−0.163 (− 0.747–0.421)	−0.466 (− 0.903–0.029)
Coeff on length of hospitalization	0.156 (− 0.368–0.680)	−0.096 (− 0.597–0.405)	−0.295 (− 0.741–0.150)	−0.381 (− 0.867–0.105)
Coeff on hospitalization costs	0.057 (− 0.261–0.376)	0.073 (− 0.287–0.433)	−0.131 (− 0.398–0.135)	−0.219 (− 0.486–0.048)
*Frail #* without sarcopenia				
OR on the probability of being admitted to hospital	1.628 (0.730–3.624)	0.708 (0.264–1.893)	1.556 (0.728–3.348)	2.217 (0.918–5.383)
Coeff on number of hospital admissions	0.043 (−1.040–1.125)	0.143 (−0.773–1.059)	0.539* (0.026–1.051)	0.133 (− 0.566–0.832)
Coeff on length of hospitalization	0.831** (0.300–1.363)	0.511* (0.055–0.966)	0.701* (0.108–1.294)	0.090 (−0.768–0.947)
Coeff on hospitalization costs	0.179 (− 0.368–0.726)	0.337 (− 0.087–0.761)	0.201 (−0.242–0.644)	0.125 (− 0.219–0.469)
*Frail #* with sarcopenia				
OR on the probability of being admitted to hospital	2.255* (1.174–4.333)	1.289* (1.043–1.675)	2.041* (1.093–3.803)	1.869** (1.199–3.094)
Coeff on number of hospital admissions	0.204 (−0.639–1.046)	0.451 (− 0.350–1.251)	0.315 (− 0.102–0.731)	0.246 (−0.277–0.769)
Coeff on length of hospitalization	0.166 (− 0.442–0.775)	0.183 (− 0.387–0.754)	0.209 (−0.250–0.667)	−0.072 (− 0.492–0.347)
Coeff on hospitalization costs	−0.050 (− 0.488–0.387)	0.106* (0.0002–0.211)	0.114 (− 0.220–0.448)	0.046* (0.00005–0.092)

95% confidence intervals in parentheses. *** *p* < 0.001, ** *p* < 0.01, * *p* < 0.05

Model 1 includes the interaction term between frailty status, measured by the Frailty Index or the Frailty Trait Score, and sarcopenia as the only independent variable. Model 2 enters sociodemographic characteristics (age and its square and gender) in addition to the interaction term from Model 1. Model 3 adds to Model 2 the comorbidity severity of individual according to the Charlson Index, which is medium-low if the Charlson Index score is 1 or 2; and high if the Charlson Index score is three or higher. Moreover, polypharmacy is also included if the daily number of drugs the subject is taking is 5 or more

**Table 6:** Log-likelihood ratio tests for each regression nested model showed in Tables 3, 4 and 5

	Cross-sectional analysis		At follow-up	
	Frailty measured with the Frailty Index	Frailty measured with the FTS5	Frailty measured with the Frailty Index	Frailty measured with the FTS5
**Regression results from** Table 3				
**M2 vs M1**	359.82***		288.96***	
**M3 vs M2**	318.80***	323.05***	294.07***	255.98**
**M4 vs M3**	358.93***	367.01***	287.63***	299.02***
**Regression results from** Table 4				
**M2 vs M1**	263.48***	269.01***	270.07***	277.27***
**M3 vs M2**	272.31***	281.09***	286.12***	292.37***
**M4 vs M3**	288.82***	295.72***	290.69***	297.99***
**Regression results from** Table 5				
**M2 vs M1**	301.98***	321.14***	318.68***	326.79***
**M3 vs M2**	310.45***	333.37***	335.09***	341.20***

*FTS5* Frailty Trait Scale 5, *M* Model

*** *p* < 0.001, ** *p* < 0.01, * *p* < 0.05

Moreover, when performing the analyses only on frail older adults (Supplementary Information Table S4), presence of sarcopenia significantly reduced length of hospitalization in the FI cross-sectional raw model but does not significantly modify any other outcome. This result would confirm the limited significance of sarcopenia as a determinant of hospital use. In case of running the analyses on older adults with sarcopenia (Supplementary Information Table S5), frailty is, in fact, significantly associated with higher hospital use, especially at follow-up, when in the full model (adjusted for age, gender, comorbidity severity and polypharmacy), being frail was associated with more number of hospitalization (FTS5), higher length of stay (FI) and hospital admission costs, which increased by 55.63% per year when the FI was used and by 45.50% in case of the FTS5, compared with non-frail individuals.

## Discussion

To our knowledge, this is the first study to demonstrate in community-dwelling older adults the costs of hospitalization to frailty and sarcopenia taking into account their interaction.

While sarcopenia increases only the likelihood of being hospitalised, frailty was also related to other outcomes such as an increase in the number of hospitalizations, in hospital stay and in healthcare costs. In addition, the interaction of having frailty and sarcopenia had an increased likelihood of hospitalization at both cross-sectional and follow-up, and increased length of stay and hospital-related costs (FTS5) at follow-up. Further segmentation of the sample revealed that could be frailty and not sarcopenia what increased healthcare expenditures. Among older adults with sarcopenia, the presence of frailty, even when adjusted for potential confounders such age, gender, polypharmacy or comorbidity, increased both the number of admissions and hospital stay and exponentially increased costs. In this line, it has been reported that those older adults with sarcopenia have two-fold risk for hospitalization and a significative higher hospital stays that those without sarcopenia, increasing in $375 the cost per person and had an annual marginal increase of $2315 [34], but in that study frailty was not assessed. However, our results might indicate that probably the frailty construct, and not the presence of sarcopenia, is what increases health economic costs in the medium/long term but more studies showing the combination of these entities are needed.

Traditionally, the allocation of healthcare resources has been made based on chronic conditions profiles. However, such approach might have been wrongly understood [13], since more recent existing evidence has pointed towards a greater burden of disability and dependency, as well as their previous stages [50]. Functional decline explains health care utilization with chronic conditions and disability [4] and hospitalization represents a major driver of total HCE [32, 51] and long-term care expenditure [52]. According to these new burdensome conditions, health economic resources should be destinated to policies or interventions in which greatest health patient benefits can be obtained [32] contributing with valuable information improving decision-making processes. Our results could certify this hypothesis, although some differences have also been observed depending on the tool with which we assess frailty.

Frailty status, and not age, has been proposed as a criterion for risk interventions in older adults [53]. As an example, frail subjects spend three times more resources than robust older adults in costs derived from surgical hospitalization and total post-operative costs at six-months [54]. Although prevalence of sarcopenia and frailty are estimated at around 10% in community-dwelling older adults [55, 56], which could be interpreted as minor rates, its estimated relationship with adverse outcomes and high associated expenditures may lead to a costly public health problem [57, 58], as our findings have also supported. Moreover, our results could confirm that in addition to the contribution of sarcopenia and frailty to the healthcare costs, different frailty construct can identify different individuals at risk of suffering adverse events. The use of frailty scales may be inclusive and not substitutable [59]. Furthermore, our results enhance the necessity of identifying frailty in people with sarcopenia [22] which may help policy makers in the efficient allocation of health care resources, being an opportunity for healthcare professionals to revert these ageing associated conditions.

### Strengths

This study has many strengths, including the large population included and the use of two relevant frailty tools previously adjusted and validated in our cohort to assess frailty. Another strength is the excellent ascertainment of the hospitalization related outcomes which are not self-reported, but extracted from clinical records, which enabled us to estimate healthcare cost expenditures associated with each available GRD.

Muscle mass has been determined using DEXA, which is the gold-standard. Moreover, the inclusion of relevant cofounders in adjusted models contributes to the increase the strength of our findings.

### Limitation

Although hospitalization has been proposed as the main health costs, we could not include other resources as physiotherapy, drugs, specialist or general practitioner visits, or diagnostic tests which are not included within the corresponding DRG.

Despite our sample size, due to the segmentation according to frailty and sarcopenia status (Supplementary Information Table S6), and the low number of hospitalized events, problems of low power to detect differences could be occurring, and subsequently, more studies should confirm our results. The results could be extended to a longer period of time in order to check whether the results obtained in the current study are consistent through a longer follow-up. Moreover, having only two points of analysis might not allow us to establish causality and, hence, the results should be interpreted with caution, suggesting associations rather than causal relationships.

## Conclusions

Demographic ageing could burden health care services. Interventions to improve frailty and sarcopenia must be promoted to prevent disability and health care pressure. Strategies for early detection and integrated multidisciplinary interventions should be promoted. Our results should be used to predict future health economic trends according to patient screening and assessment.

## Supplementary Information

**Additional file 1: Table S1.** Toledo Study of Health Ageing Frailty Index. **Table S2.** Frailty Trait Scale 5 (FTS5) scoring. **Table S3.** Results on the association between the interaction term between frailty and sarcopenia and the hospitalization-related outcomes, both at the cross-sectional level and at-follow up. **Table S4.** Results on the association between sarcopenia and the hospitalization-related outcomes, both at the cross-sectional level and at-follow up, only on the frail older adults. **Table S5.** Results on the association between frailty and the hospitalization-related outcomes, both at the cross-sectional level and at-follow up, only on the older adults with sarcopenia. **Table S6.** Cross-tabulations of sarcopenia and frailty according to the FTS5 and the FI.

## Data Availability

Data will be available upon request to the corresponding author.

## Footnotes

1 Since the number of hospital admissions and the average length of stay do not allow for zero-count data, a zero-truncated regression model was run for the second part of the two-part model for these outcomes. The data are truncated because there are no observations on individuals who stayed for zero days nor for negative values [46]. Additionally, we used robust standard errors for the parameter estimates.

2 The coefficients on hospitalization costs in the text do not match the coefficients reported in the tables, due to the corresponding exponential transformation associated with the log-link. For example, the 22.85% comes from the calculation of exp. (0.2058307)-1.

3 When the interaction between frailty and sarcopenia was included in addition to each of these terms individually, the results showed that none of the interactions between frailty and sarcopenia were statistically significant, but for its interaction in the full model when assessing hospitalization costs and frailty was measured through the FTS5 (Table S3, Supplementary Material).
