# Supplementary material for: Healthcare cost expenditures associated to frailty and sarcopenia
Source: BMC Geriatr. 2022 Sep 13;22:747. doi: 10.1186/s12877-022-03439-z (PMC9469617; doi:10.1186/s12877-022-03439-z)
Supplement: Supplementary file 1 — Additional file 1: Table S1. Toledo Study of Health Ageing Frailty Index. Table S2. Frailty Trait Scale 5 (FTS5) scoring. Table S3. Results on the association between the interaction term between frailty and sarcopenia and the hospitalization-related outcomes, both at the cross-sectional level and at-follow up. Table S4. Results on the association between sarcopenia and the hospitalization-related outcomes, both at the cross-sectional level and at-follow up, only on the frail older adults. Table S5. Results on the association between frailty and the hospitalization-related outcomes, both at the cross-sectional level and at-follow up, only on the older adults with sarcopenia. Table S6. Cross-tabulations of sarcopenia and frailty according to the FTS5 and the FI. [file 12877_2022_3439_MOESM1_ESM.docx]

Supplementary Information

Table S1. Toledo Study of Health Ageing Frailty Index.

|  | Rockwood Frailty Index | TSHA Frailty Index | Score |
| --- | --- | --- | --- |
| 1. | Help bathing | Same | Yes =1, No =0 |
| 2. | Help dressing | Same | Yes =1, No =0 |
| 3. | Help getting in/out of chair and bed | Same | Yes =1, No =0 |
| 4. | Help walking a round house | Same | Yes =1, No =0 |
| 5. | Help eating | Same | Yes =1, No =0 |
| 6. | Help grooming | Same | Yes =1, No =0 |
| 7. | Help using toilet | Same | Yes =1, No =0 |
| 8. | Help up stairs | Same | Yes =1, No =0 |
| 9. | Help lifting 10 lbs | Same | Yes =1, No =0 |
| 10. | Help shopping | Same | Yes =1, No =0 |
| 11. | Help with housework | Same | Yes =1, No =0 |
| 12. | Help with meal preparations | Same | Yes =1, No =0 |
| 13. | Help taking medication | Same | Yes =1, No =0 |
| 14. | Help with finances | Same | Yes =1, No =0 |
| 15. | Lost more than 10 lbs in the last year | Same | Yes =1, No =0 |
| 16. | Selfrating of health | Same | Very poor =1, Poor =0.75, Regular =0.5, Good =0.25, Very good =0 |
| 17. | How health has changed in last year | Same | Much better =0, Better =0, Same =0, Worse =1, Much worse =1 |
| 18. | Stayed in bed at least half the day due to health (in las month) | During the past moth how often have yo gone or has visited the doctor for being | Any time =0, Once =0.5, Twice or more =1 |
| 19. | Cut down on usual activity (in the last month): Activity. Worst quintile of PASE | Activity (Physical Activity Scale for the Elderly, PASE) | Worst quintile =1, Rest =0 |
| 20. | Walk outside | Same | Any time =1, Once or twice=0.5, Three times or more =0 |
| 21. | Feel everything is an effort | Same | Most of time =1, Some time =0.5, Rarely =0 |
| 22. | Feel depressed | Feel depressed or anxious | Not =0, Moderately =0.5, Very =1 |
| 23. | Feel happy | Same | Very or pretty happy =0, neither happy nor unhappy =0.5, Very o rather unhappy =1 |
| 24. | Feel lonely | Can you talk about your problems with someone close at least your family and friends | Never or almost never =1, Sometimes =0.5, Mostly or always =0 |
| 25. | Have trouble getting going | Do you feel you can do anything you propose? | Never or almost never =1, Sometimes =0.5, Mostly or always =0 |
| 26. | High blood pressure | Same | Yes =1, Suspect =0.5, No =0 |
| 27. | Heart attack | Same | Yes =1, Suspect =0.5, No =0 |
| 28. | CHF | Same | Yes =1, Suspect =0.5, No =0 |
| 29. | Stroke | Same | Yes =1, Suspect =0.5, No =0 |
| 30. | Cancer | Same | Yes =1, Suspect =0.5, No =0 |
| 31. | Diabetes | Same | Yes =1, Suspect =0.5, No =0 |
| 32. | Arthritis | Same | Yes =1, Suspect =0.5, No =0 |
| 33. | Chronic lung disease | Same | Yes =1, Suspect =0.5, No =0 |
| 34. | MMSE | Same | <10= 1, 11-17=0.75, 18-20= 0.5, 21-24=0.25, >24 =0 |
| 35. | Peak Flow | Forced expiratory volume in 1 second (FEV) (litres) | <1.39 |
| 36. | Shoulder strength | Same | Men <10.84 =1, Women<6.4 =1, Other values =0 |
| 37. | BMI | Same | <18.5 or ≥30 =1, 25 -<30 =0.5, other values =0 |
| 38. | Grip strength | Same | Worst BMI quintil by sex =1, Other value =0 |
| 39. | Usual pace | Same | Worst height quintil by sex =1, Other value =0 |
| 40. | Rapid pace | Balance | 1= side by side 0-9 sec or unable, other value =0 |

FEV: Forced expiratory volume. PASE: Physical Activity Scale for the Elderly.

Table S2. Frailty Trait Scale 5 (FTS5) scoring.

| Score | BMI, kg/m^2^ | | PASE | Gait Speed ^a^ | Grip strength, kg | |  | Progressive Romberg | | |
| --- | --- | --- | --- | --- | --- | --- | --- | --- | --- | --- |
|  |  |  |  |  |  |  |  |  |  |  |
|  |  |  |  |  | Women | Men |  | Score | Position Seconds | Seconds |
| 0 | 23.01-26.99 |  | >194 | <2.45 | >22 | >29 |  | 0 | Tandem | ≤10 |
| 1 | 27-28.99 | 21.01-23 | 174.61-194 | 2.45-2.99 | 19.81-22 | 26.11-29 |  | 2.5 | Tandem | 3.01-9.99 |
| 2 | 29-30.99 | 19.01-21 | 155.21-174.6 | 3.00-3.54 | 17.61-19.8 | 23.21-26.1 |  | 5 | Tandem | ≤3 |
| 3 | 31-32.99 | 17.01-19 | 135.81-155.2 | 3.55-4.09 | 15.41-17.6 | 20.31-23.2 |  |  | Semitandem | ≤10 |
| 4 | 33-34.99 | 15.01-17 | 116.41-135.8 | 4.10-4.64 | 13.21-15.4 | 17.41-20.3 |  | 7.5 | Semitandem | <10 |
| 5 | 35-36.99 | 13.01-15^b^ | 97.01-116.4 | 4.65-5.19 | 11.01-13.2 | 14.51-17.4 |  |  | Side by side | ≤10 |
| 6 | 37-38.99 | 11.01-13^b^ | 77.61-97 | 5.20-5.74 | 8.81-11.0 | 11.61-14.5 |  | 10 | Side by side | <10 |
| 7 | 39-40.99 | NA | 58.21-77.6 | 5.75-6.29 | 6.61-8.8 | 8.71-11.6 |  |  |  |  |
| 8 | 41-42.99 | NA | 38.81-58.2 | 6.30-6.84 | 4.41-6.6 | 5.81-8.7 |  |  |  |  |
| 9 | 43-44.99 | NA | 19.41-38.8 | 6.85-7.39 | 2.21-4.4 | 2.91-5.8 |  |  |  |  |
| 10 | ≤45 | NA | 0-19.4 | ≤7.4 | 0-2.2 | 0-2.9 |  |  |  |  |

BMI: Body Mass Index. PASE: Physical Activity Scale for the Elderly.

FTS_5_ includes all the items of the table (range 0-50), and frail participants are those with FTS_5_ scores >25.

^a^: Gait speed refers to time in accomplish 3-metres at usual pace in seconds.

^b^: Model estimation.Table S3. Results on the association between the interaction term between frailty and sarcopenia and the hospitalization-related outcomes, both at the cross-sectional level and at-follow up

|  | **Cross-sectional analysis** | | **At follow-up** | |
| --- | --- | --- | --- | --- |
|  | Frailty measured with the Frailty Index | Frailty measured with the FTS5 | Frailty measured with the Frailty Index | Frailty measured with the FTS5 |
| Model 1 |  |  |  |  |
| *Having sarcopenia* |  |  |  |  |
| OR on the probability of being admitted to hospital | 1.318  (0.934 – 1.862) | 1.395  (0.957 – 2.034) | 1.365*  (1.005 – 1.854) | 1.292  (0.916 – 1.822) |
| Coeff on number of hospital admissions | 0.258  (-0.443 – 0.959) | 0.101  (-0.683 – 0.884) | -0.095  (-0.618 – 0.429) | -0.335  (-0.728 – 0.059) |
| Coeff on length of hospitalization | 0.264  (-0.185 – 0.713) | 0.007  (-0.453 – 0.466) | -0.125  (-0.487 – 0.238) | -0.122  (-0.527 – 0.282) |
| Coeff on hospitalization costs | -0.005  (-0.327 – 0.317) | 0.043  (-0.341 – 0.427) | -0.096  (-0.377 – 0.185) | -0.177  (-0.429 – 0.075) |
| *Being frail* |  |  |  |  |
| OR on the probability of being admitted to hospital | 2.851**  (1.333 – 6.101) | 1.301  (0.520 – 3.256) | 2.698**  (1.298 – 5.608) | 3.092**  (1.454 – 6.575) |
| Coeff on number of hospital admissions | 0.391  (-0.694 – 1.477) | 0.467  (-0.311 – 1.245) | 0.733**  (0.241 – 1-226) | 0.165  (-0.561 – 0.891) |
| Coeff on length of hospitalization | 1.302***  (0.745 – 1.859) | 0.891**  (0.261 – 1.521) | 0.985**  (0.371 – 1.599) | 0.337  (-0.510 – 1.184) |
| Coeff on hospitalization costs | 0.380  (-0.240 – 1.001) | 0.362  (-0.070 – 0.795) | 0.259  (-0.207 – 0.726) | -0.045  (-0.579 – 0.490) |
| *Frail # with sarcopenia* |  |  |  |  |
| OR on the probability of being admitted to hospital | 0.930  (0.346 – 2.499) | 1.026  (0.355 – 2.965) | 0.693  (0.267 – 1.800) | 0.505  (0.205 – 1.244) |
| Coeff on number of hospital admissions | -0.418  (-1.874 – 1.039) | -0.162  (-1.423 – 1.099) | -0.196  (-0.977 – 0.586) | 0.476  (-0.460 – 1.412) |
| Coeff on length of hospitalization | -1.142  (-2.323 – 0.039) | -0.462  (-1.331 – 0.407) | -0.402  (-1.193 – 0.388) | -0.114  (-1.098 – 0.871) |
| Coeff on hospitalization costs | -0.380  (-1.210 – 0.449) | 0.150  (-0.468 – 0.768) | -0.071  (-0.676 – 0.534) | 0.279  (-0.390 – 0.948) |
| Model 2 |  |  |  |  |
| *Having* *sarcopenia* |  |  |  |  |
| OR on the probability of being admitted to hospital | 1.361  (0.928 – 1.994) | 1.450  (0.960 – 2.189) | 1.666**  (1.178 – 2.356) | 1.615*  (1.108 – 2.355) |
| Coeff on number of hospital admissions | 0.567  (-0.340 – 1.474) | 0.401  (-0.579 – 1.380) | -0.100  (-0.737 – 0.536) | -0.340  (-0.795 – 0.115) |
| Coeff on length of hospitalization | 0.314  (-0.229 – 0.857) | 0.034  (-0.479 – 0.547) | -0.203  (-0.661 – 0.255) | -0.211  (-0.691 – 0.269) |
| Coeff on hospitalization costs | 0.078  (-0.248 – 0.405) | 0.111  (-0.271 – 0.493) | -0.085  (-0.374 – 0.203) | -0.158  (-0.425 – 0.109) |
| *Being frail* |  |  |  |  |
| OR on the probability of being admitted to hospital | 2.154  (0.963 – 4.821) | 0.910  (0.348 – 2.379) | 2.027  (0.937 – 4.389) | 2.645*  (1.129 – 6.200) |
| Coeff on number of hospital admissions | 0.416  (-0.823 – 1.655) | 0.363  (-0.778 – 1.504) | 0.687*  (0.135 – 1.240) | 0.321  (-0.410 – 1.053) |
| Coeff on length of hospitalization | 1.256***  (0.716 – 1.796) | 0.945**  (0.380 – 1-510) | 0.918**  (0.250 – 1.585) | 0.348  (-0.538 – 1.235) |
| Coeff on hospitalization costs | 0.379  (-0.205 – 0.962) | -0.258  (-0.648 – 0.131) | 0.312  (-0.139 – 0.763) | 0.100  (-0.410 – 0.610) |
| *Frail # with sarcopenia* |  |  |  |  |
| OR on the probability of being admitted to hospital | 1.025  (0.367 – 2.860) | 1.256  (0.423 – 3.731) | 0.739  (0.274 – 1.994) | 0.525  (0.197 – 1.400) |
| Coeff on number of hospital admissions | -0.465  (-2.015 – 1.084) | -0.082  (-1.595 – 1.430) | -0.149  (-0.965 – 0.666) | 0.334  (-0.565 – 1.233) |
| Coeff on length of hospitalization | -1.142  (2.316 – 0.032) | -0.578  (-1.423 – 0.266) | -0.344  (-1.164 – 0.475) | -0.124  (-1.146 – 0.897) |
| Coeff on hospitalization costs | -0.343  (-1.112 – 0.426) | 0.094  (-0.490 – 0.677) | -0.060  (-0.626 – 0.507) | 0.149  (-0.454 – 0.752) |
| Model 3 |  |  |  |  |
| *Having* *sarcopenia* |  |  |  |  |
| OR on the probability of being admitted to hospital | 1.199  (0.812 – 1.769) | 1.265  (0.836 – 1.915) | 1.477*  (1.040 – 2.098) | 1.452  (0.989 – 2.132) |
| Coeff on number of hospital admissions | 0.401  (-0.437 – 1.240) | 0.255  (-0.664 – 1.174) | -0.163  (-0.747 – 0.421) | -0.466  (-0.943 – 0.011) |
| Coeff on length of hospitalization | 0.156  (-0.368 – 0.680) | -0.096  (-0.597 – 0.405) | -0.295  (-0.741 – 0.150) | -0.381  (-0.866 – 0.105) |
| Coeff on hospitalization costs | 0.057  (-0.261 – 0.376) | 0.073  (-0.248 – 0.394) | -0.131  (-0.398 – 0.135 | -0.219  (-0.498 – 0.059) |
| *Being frail* |  |  |  |  |
| OR on the probability of being admitted to hospital | 1.562  (0.700 – 3.488) | 0.678  (0.255 – 1.806) | 1.508  (0.701 – 3.246) | 2.120  (0.931 – 4.828) |
| Coeff on number of hospital admissions | 0.043  (-1.040 – 1.125) | 0.143  (-0.773 – 1.059) | 0.539*  (0.026 – 1.051) | 0.133  (-0.566 – 0.832) |
| Coeff on length of hospitalization | 0.831**  (0.300 – 1.363) | 0.511*  (0.055 – 0.966) | 0.701*  (0.108 – 1.294) | 0.090  (-0.768 – 0.947) |
| Coeff on hospitalization costs | 0.179  (-0.368 – 0.726) | -0.337  (-1.115 – 0.442) | 0.201  (-0.242 – 0.644) | -0.012  (-0.531 – 0.506) |
| *Frail # with sarcopenia* |  |  |  |  |
| OR on the probability of being admitted to hospital | 1.266  (0.452 – 3.544) | 1.592  (0.525 – 4.822) | 0.870  (0.324 – 2.335) | 0.595  (0.229 – 1.551) |
| Coeff on number of hospital admissions | -0.240  (-1.690 – 1.209) | 0.052  (-1.261 – 1.366) | -0.061  (-0.825 – 0.703) | 0.578  (-0.325 – 1.481) |
| Coeff on length of hospitalization | -0.821  (-1.637 – 1.363) | -0.231  (-1.000 – 0.538) | -0.197  (-0.959 – 0.565) | 0.219  (-0.785 – 1.224) |
| Coeff on hospitalization costs | -0.287  (-0.999 – 0.426) | 0.158  (-0.714 – 1.030) | 0.044  (-0.497 – 0.586) | 0.278  (-0.346 – 0.902) |

95% confidence intervals in parentheses. *** p<0.001, ** p<0.01, * p<0.05

Model 1 includes the interaction term between frailty status, measured by the Frailty Index or the Frailty Trait Score, and sarcopenia, in addition to each of them individually as the only independent variables. Model 2 enters sociodemographic characteristics (age and its square and gender) in addition to the interaction term from Model 1. Model 3 adds to Model 2 the comorbidity severity of individual according to the Charlson Index, which is medium-low if the Charlson Index score is 1 or 2; and high if the Charlson Index score is three or higher. Moreover, polypharmacy is also included if the daily number of drugs the subject is taking is 5 or more.

Table S4. Results on the association between sarcopenia and the hospitalization-related outcomes, both at the cross-sectional level and at-follow up, **only on the frail older adults**

|  | **Cross-sectional analysis** | | **At follow-up** | |
| --- | --- | --- | --- | --- |
|  | **Sarcopenia if frailty is measured via the Frailty Index** | **Sarcopenia if frailty is measured via the FTS5** | **Sarcopenia if frailty is measured via the Frailty Index** | **Sarcopenia if frailty is measured via the FTS5** |
| **Model 1** |  |  |  |  |
| OR on the probability of being admitted to hospital | 1.226  (0.483 – 3.113) | 1.431  (0.529 – 3.872) | 0.946  (0.381 – 2.347) | 0.652  (0.282 – 1.506) |
| Coeff on number of hospital admissions | -0.159  (-1.454 – 1.135) | -0.062  (-1.061 – 0.938) | -0.290  (-0.878 – 0.297) | 0.142  (-0.714 – 0.997) |
| Coeff on length of hospitalization | 0.878*  (0.149 – 1.608) | -0.455  (-1.202 – 0.292) | -0.527  (-1.238 – 0.184) | -0.236  (-1.140 – 0.668) |
| Coeff on hospitalization costs | -0.385  (-1.160 – 0.390) | 0.193  (-0.297 – 0.683) | -0.167  (-0.710 – 0.376) | 0.102  (-0.522 – 0.727) |
| **Model 2** |  |  |  |  |
| OR on the probability of being admitted to hospital | 1.301  (0.455 – 3.717) | 2.208  (0.747 – 6.529) | 1.103  (0.397 – 3.065) | 0.937  (0.372 – 2.365) |
| Coeff on number of hospital admissions | -0.244  (-1.442 – 0.955) | 0.467  (-1.660 – 2.593) | -0.308  (-0.895 – 0.279) | 0.083  (-0.824 – 0.991) |
| Coeff on length of hospitalization | 0.862*  (0.185 – 1.539) | -0.413  (-1.493 – 0.667) | -0.543  (-1.297 – 0.211) | -0.290  (-1.370 – 0.790) |
| Coeff on hospitalization costs | -0.502  (-1.266 – 0.263) | 0.325  (-0.299 – 0.948) | -0.280  (-0.737 – 0.178) | -0.022  (-0.602 – 0.558) |
| **Model 3** |  |  |  |  |
| OR on the probability of being admitted to hospital | 1.551  (0.521 – 4.616) | 2.497  (0.869 – 7.174) | 1.091  (0.393 – 3.029) | 0.933  (0.370 – 2.350) |
| Coeff on number of hospital admissions | -0.813  (-1.702 – 0.077) | 0.470  (-0.971 – 1.911) | -0.271  (-0.779 – 0.236) | 0.210  (-0.608 – 1.027) |
| Coeff on length of hospitalization | 0.813*  (0.124 – 1.503) | -0.331  (-1.251 – 0.590) | -0.437  (-1.023 – 0.149) | -0.149  (-1.053 – 0.755) |
| Coeff on hospitalization costs | -0.480  (-1.165 – 0.206) | 0.293  (-0.258 – 0.844) | -0.234  (-0.661 – 0.193) | 0.092  (-0.463 – 0.647) |

95% confidence intervals in parentheses. *** p<0.001, ** p<0.01, * p<0.05.

Model 1 includes sarcopenia as the only independent variable. Model 2 enters sociodemographic characteristics (age and its square and gender) in addition to sarcopenia. Model 3 adds to Model 2 the comorbidity severity of individual according to the Charlson Index, which is medium-low if the Charlson Index score is 1 or 2; and high if the Charlson Index score is three or higher. Moreover, polypharmacy is also included if the daily number of drugs the subject is taking is 5 or more.

Table S5. Results on the association between frailty and the hospitalization-related outcomes, both at the cross-sectional level and at-follow up, **only on the older adults with sarcopenia**

|  | **Cross-sectional analysis** | | **At follow-up** | |
| --- | --- | --- | --- | --- |
|  | **Frailty measured with the Frailty Index** | **Frailty measured with the FTS5** | **Frailty measured with the Frailty Index** | **Frailty measured with the FTS5** |
| **Model 1** |  |  |  |  |
| OR on the probability of being admitted to hospital | 2.651**  (1.408 – 4.991) | 1.334  (0.782 – 2.277) | 1.870*  (1.013 – 3.454) | 1.561  (0.951 – 2.560) |
| Coeff on number of hospital admissions | -0.026  (-1.002 – 0.950) | 0.305  (-0.692 – 1.302) | 0.538  (-0.072 – 1.147) | 0.641*  (0.048 – 1.234) |
| Coeff on length of hospitalization | 0.160  (-0.483 – 0.802) | 0.429  (-0.173 – 1.031) | 0.583*  (0.083 – 1.082) | 0.223  (-0.280 – 0.727) |
| Coeff on hospitalization costs | 0.001  (-0.553 – 0.553) | -0.212  (-0.656 – 0.231) | 0.189  (-0.199 – 0.576) | 0.234  (-0.170 – 0.638) |
| **Model 2** |  |  |  |  |
| OR on the probability of being admitted to hospital | 2.314*  (1.175 – 4.559) | 1.181  (0.682 – 2.045) | 1.583  (0.820 – 3.056) | 1.468  (0.864 – 2.493) |
| Coeff on number of hospital admissions | -0.019  (-1.077 – 1.040) | 0.447  (-0.462 – 1.356) | 0.684  (-0.004 – 1.371) | 0.692*  (0.145 – 1.240) |
| Coeff on length of hospitalization | 0.117  (-0.619 – 0.853) | 0.416  (-0.151 – 0.983) | 0.637*  (0.104 – 1.170) | 0.237  (-0.231 – 0.705) |
| Coeff on hospitalization costs | 0.126  (-0.381 – 0.634) | 0.123*  (0.013 – 0.233) | 0.443*  (0.101 – 0.786) | 0.352*  (0.016 – 0.688) |
| **Model 3** |  |  |  |  |
| OR on the probability of being admitted to hospital | 2.261*  (1.129 – 4.526) | 1.167  (0.659 – 2.065) | 1.348  (0.688 – 2.642) | 1.296  (0.743 – 2.261) |
| Coeff on number of hospital admissions | -0.131  (-1.047 – 0.785) | 0.474  (-0.498 – 1.446) | 0.600  (-0.144 – 1.344) | 0.756**  (0.224 – 1.289) |
| Coeff on length of hospitalization | 0.112  (-0.570 – 0.795) | 0.322  (-0.251 – 0.896) | 0.587*  (0.082 – 1.091) | 0.274  (-0.202 – 0.751) |
| Coeff on hospitalization costs | 0.051  (-0.423 – 0.526) | 0.046  (-0.431 – 0.531) | 0.442*  (0.091 – 0.794) | 0.375*  (0.039 – 0.711) |

95% confidence intervals in parentheses. *** p<0.001, ** p<0.01, * p<0.05

Model 1 includes frailty status, measured by the Frailty Index or the Frailty Trait Score, as the only independent variable. Model 2 enters sociodemographic characteristics (age and its square and gender) in addition to frailty. Model 3 adds to Model 2 the comorbidity severity of individual according to the Charlson Index, which is medium-low if the Charlson Index score is 1 or 2; and high if the Charlson Index score is three or higher. Moreover, polypharmacy is also included if the daily number of drugs the subject is taking is 5 or more.

Table S6. Cross-tabulations of sarcopenia and frailty according to the FTS5 and the FI.

|  | | Sarcopenia | | Total |
| --- | --- | --- | --- | --- |
|  |  | Without Sarcopenia | With Sarcopenia |  |
| Frailty, according to the FTS5 | No frail | 1,027 | 190 | 1,217 |
|  | Frail | 28 | 114 | 142 |
| Frailty, according to the FI | No frail | 1,025 | 251 | 1,276 |
|  | Frail | 30 | 53 | 83 |
| Total | | 1,055 | 304 | 1,359 |

FTS5: Frailty Trait Scale 5. FI: Frailty Index.
